# Long-term effects of air pollution on the incidence and progression of multiple sclerosis: A population cohort study in Isfahan, Iran

**DOI:** 10.1371/journal.pone.0327635

**Published:** 2025-08-08

**Authors:** Omid Mirmosayyeb, Saeed Vaheb, Alireza Afshari-Safavi, Aysa Shaygannejad, Mohammad Ali Sahraian, Ali Atamaleki, Sharareh Eskandarieh, Vahid Shaygannejad

**Affiliations:** 1 Isfahan Neurosciences Research Center, Isfahan University of Medical Sciences, Isfahan, Iran; 2 Department of Neurology, School of Medicine, Isfahan University of Medical Sciences, Isfahan, Iran; 3 Department of Biostatistics and Epidemiology, Faculty of Health, North Khorasan University of Medical Sciences, Bojnurd, Iran; 4 Multiple Sclerosis Research Center, Neuroscience Institute, Tehran University of Medical Sciences, Tehran, Iran; 5 Department of Environmental Health Engineering, School of Public Health, North Khorasan University of Medical Sciences, Bojnurd, Iran; Shahrekord University of Medical Science, IRAN, ISLAMIC REPUBLIC OF

## Abstract

**Background:**

Multiple sclerosis (MS) is a neurodegenerative disorder of the central nervous system characterized by autoimmune inflammation. Recent research indicates that environmental factors, particularly air pollution, may significantly affect the risk of developing MS.

**Objective:**

This study investigates the association between PM_2.5_ levels, as a measure of air pollution, and the incidence of MS in Isfahan, Iran, a city with one of the highest reported MS prevalence rates in the country.

**Methods:**

A retrospective population-based cohort study was conducted using data from the National MS registry of Iran and Isfahan’s air pollution monitoring department from 2011 to 2021. The incidence of MS across urban areas was calculated, and the relationship between PM_2.5_ levels and the incidence rate ratio (IRR) of MS was assessed using a Poisson generalized regression models.

**Results:**

PM_2.5_ levels averaged 41.99 µg/m^3^ across the study period (first year: 59.21 ± 33.56; mid-study: 30.51 ± 11.77; final year: 37.71 ± 53.64), persistently exceeding safety standards. Three-year cumulative exposure showed significant association with higher MS incidence (IRR = 1.027, 95%CI = 1.022–1.031, p < 0.001) and correlated with disease progression in progressive MS cases.

**Conclusion:**

Long-term exposure to PM_2.5_ is associated with an increased incidence of MS and disease progression, emphasizing the critical need for improved air quality management strategies.

## 1. Introduction

Multiple sclerosis (MS) is a neurodegenerative disorder of the central nervous system (CNS) caused by chronic autoimmune inflammation [1]. This condition is known by periods of relapse and remission. During relapse, inflammatory factors often cross the blood-brain barrier (BBB) and reactivate autoaggressive T cells [2]. Consequently, this inflammation may can cause demyelination, neurodegeneration, and BBB destruction, leading to various symptoms depending on the lesion’s location [3].

Although the exact etiology of MS is still unknown, research has shown that genetic predisposition [4], specific viral infections [5], vitamin D deficiency [6], and obesity during puberty [7] can contribute to its pathogenesis. Recently, there has been increased attention to environmental risk factors, such as air pollution, which may influence the risk of MS [8].

Air pollution, defined by the World Health Organization (WHO) as an increase in the level of suspended aerodynamic diameter less than 2.5 micrometers (PM_2.5_) [9,10], can have general consequences for public health. Environmental Protection Agency (EPA) provides classification thresholds for PM_2.5_ concentrations, which can serve as a complementary reference when evaluating pollution levels [11].

PM_2.5_ can carry various toxic substances. Unlike PM_10_, which ranges from 2 to 10 micrometers, PM_2.5_ particles are small enough to bypass the mucosal barrier of the respiratory system and potentially enter the bloodstream [12,13]. These particles can increase oxidative stress, exacerbate immune responses, and increase the risk of autoimmune disorders [14,15]. Evidence suggests that PM_2.5_ may play a role in neuroinflammatory processes in various disorders such as Alzheimer’s and Parkinson’s by causing systemic inflammation, disrupting the blood-brain barrier, and activating microglia [16]. Major sources of PM_2.5_ include combustion engines, industrial activities, volcanic eruptions, and forest fires, impacting both the troposphere and stratosphere [17,18].

Previous studies have examined the link between air pollution, and the risk of MS. Angelici et al. found that the relapse rate in People with MS (PwMS) increased by 40% on the most polluted days of the year compared to days with the lowest levels of air pollution [19]. Similarly, Ashtari et al. highlighted a correlation between higher air pollution levels and an increased risk of relapse [20]. Additionally, Tateo et al. identified PM_2.5_ as a significant risk factor for exacerbating MS symptoms [9]. However, studies examining the short-term effects of pollution on MS incidence [19,21] did not show significant results in this context. Furthermore, recent studies have reported a strong association between long-term exposure to PM_2.5_ and increased MS prevalence [22], as well as a higher risk of relapse following short-term exposure to elevated PM_2.5_ levels, particularly among younger individuals [23].

Given the conflicting results of previous studies, a comprehensive study with large sample size and long-term follow-up is essential to clarify the potential impact of air pollution on the annual incidence of MS.

Isfahan, is ideal for this study for two main reasons. First, previous studies indicate that Isfahan has one of the highest MS rates in Iran and the Middle East [24,25], although the cause of this high prevalence remains unclear. Second, Isfahan experiences significant air pollution from major industrial activities by companies like large steel and petrochemical industries, along with heavy traffic and a dense population. As a result, Isfahan is among the most polluted cities in the Iran [26,27]. Considering these conditions, the question arises: Is there a connection between this level of air pollution and the high prevalence of MS in Isfahan? This research will examine the annual incidence of MS in different urban areas of Isfahan and analyze its associations with daily PM_2.5_ levels. Additionally, this study aimed to investigate the relationship between the severity of air pollution and the progression of MS.

## 2. Method

This retrospective population-based cohort study utilized data from the Statistical Center of the Islamic Republic of Iran, the Vice-Chancellor of Treatment of the Medical University of Isfahan (MUI) [25], National MS Registry of Iran (NMSRI) – a nationwide database employing the 2017 McDonald criteria with mandatory reporting by all neurology centers [28], and the air pollution monitoring department of the Isfahan Environmental Protection Organization [27] from 2011 to 2021. The data for this study were collected between August 2023 and August 2024 using information obtained from these sources.

### 2.1. Study location

Iran is the second-most populated country in the Middle East and the 20th-most populated country in the world. Isfahan, one of Iran’s most populated provinces, is located at latitude 30–34 degrees N and longitude 49–55 degrees E, in an area of 551 km^2^. The city of Isfahan, comprising 15 urban areas, had a population of 1,996,443 in 2020, according to the latest estimate by the Iranian Statistics Center [25].

The Iranian Statistics Center conducts a national population census every ten years and estimates population figures for the intervening years based on annual birth and death rates from 1995 to 2010 and 2020. Based on these estimates, the Isfahan Municipality announces the city’s annual population.

### 2.2. Air pollution monitoring centers

Isfahan has seven 24-hour air pollution monitoring centers that report atmospheric pollutants, including PM_2.5_. The results from these centers are sent monthly to the Environmental Protection Center of Isfahan province, and this study was designed using these data. The seven monitoring centers are nearly homogeneously distributed across the city: two in the northeast (NE), two in the northwest (NW), two in the southeast (SE), and one in the southwest (SW).

Urban regions 1, 2, 3, 8, 11, and 12 are in the NW; regions 4, 6, 14, and 15 are in the SE; regions 7 and 10 are in the NE; and regions 5, 9, and 13 are in the SW. The final pollution levels for all four urban areas (NE, NW, SW, and SE) were estimated based on the average results from the respective air pollution monitoring centers.

### 2.3. Data source

**Fig 1:**
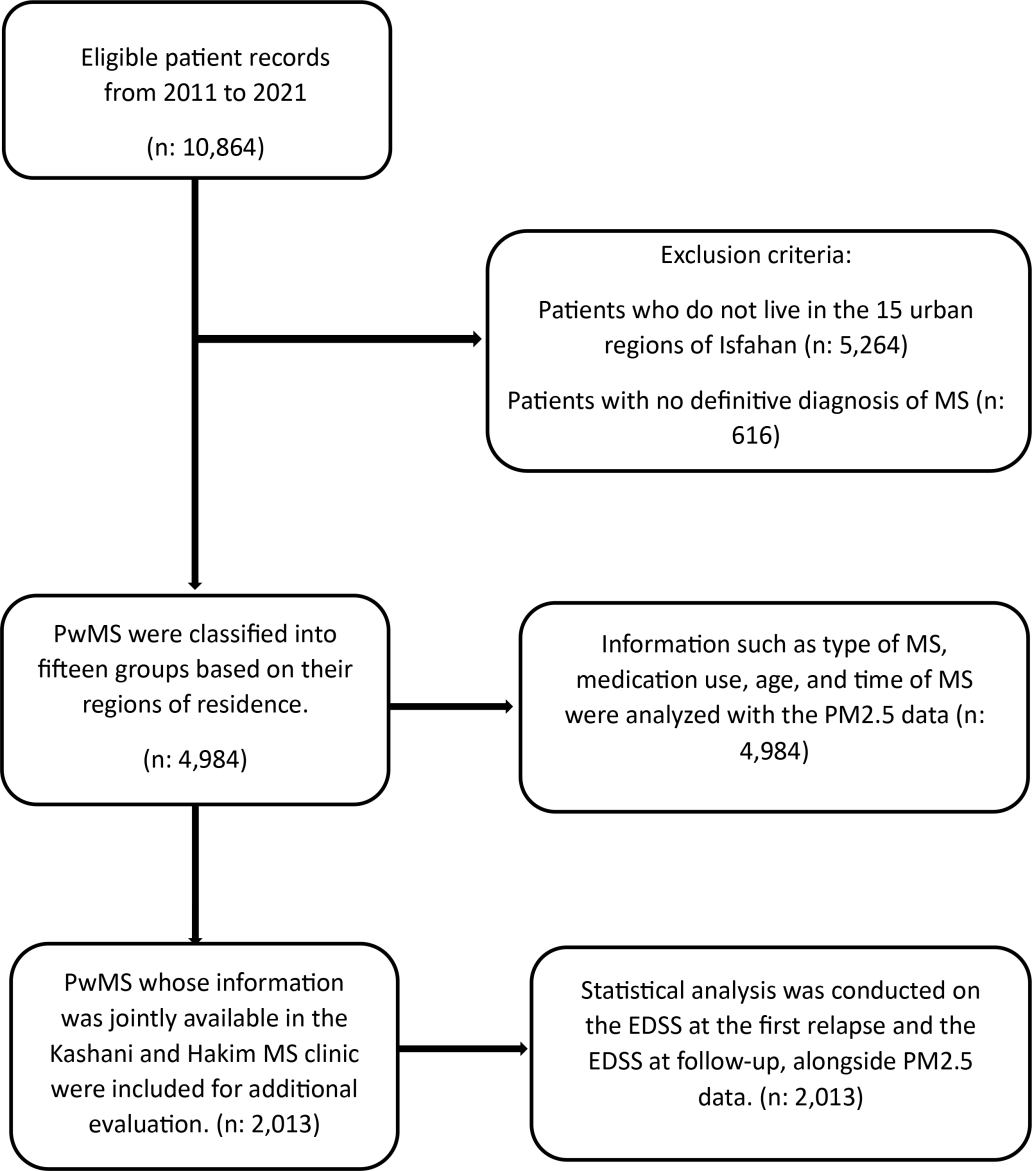
Flow diagram of the study process and participant selection.

The information for the PwMS under study was obtained from the Vice-Chancellor of Treatment of MUI and NMSRI. This data included the type of MS, the medications used, the year of disease onset, the patients’ ages, and their addresses. All PwMS in the database had been definitively diagnosed with MS by a neurologist based on the McDonald criteria [29–31],and each diagnosis was subsequently reviewed and confirmed by a specialized expert commission before being included in the database. Patients residing outside the 15 urban regions of Isfahan or those who had not been definitively diagnosed with MS were excluded from the study. PwMS living in Isfahan City were classified into fifteen groups based on their municipal regions. The annual incidence of MS in each urban region was calculated by dividing the number of new-onset patients in that region by its estimated population. Additional data, including the Expanded Disability Status Scale (EDSS) at the first relapse and during follow-up, were obtained from the databases of the Kashani MS Clinic and Hakim MS Clinic [32]. For more information, refer to the study flowchart (Fig 1).

The association between PM_2.5_ levels and MS incidence was measured using reports from 24-hour air pollution monitoring centers and the annual incidence of MS in each region.

### 2.4. Ethical approval and compliance

The ethical committee of Isfahan University of Medical Sciences approved this study (Ethical code: IR.MUI.MED.REC.1399.055). The research was conducted following the principles of the Declaration of Helsinki and its subsequent revisions. All data from the NMSRI were gathered with patient consent, and this study utilized coded data that excluded personal identification information.

### 2.5. Statistical analysis

The characteristics of the study participants were reported by frequency (percentage), mean (standard deviation), and median (interquartile range) using a variable scale.

The annual incidence of MS per 10,000 populations was calculated for four areas (NE, NW, SE, and SW) and compared through a chi-square test. The Kruskal-Wallis test was also used to compare the annual average of PM_2.5_ between the four areas. The correlation between PM_2.5_ and Delta EDSS (Current EDSS-First EDSS) was evaluated through Spearman’s coefficient.

We investigated the association between MS incidence and PM_2.5_ exposure using Poisson generalized regression models analyzing three exposure windows:

Model I (non-delayed): Annual mean PM_2.5_ in diagnosis year
Model II (1-year lag): Annual mean PM_2.5_ one year before diagnosis
Model III (3-year cumulative): Mean PM_2.5_ over three preceding years (t-1 to t-3), calculated as:
Cumulative PM_2.5_ = (PM_2.5__t-1 + PM_2.5__t-2 + PM_2.5__t-3)/3

All models produced incidence rate ratios (IRR) with 95% confidence intervals and two-tailed p-values, with statistical significance set at α = 0.05. The IRRs were calculated by dividing the incidence rate of MS in areas with higher PM_2.5_ exposure by the incidence rate in areas with lower exposure, using Poisson regression models adjusted for population size. All analyses were conducted using IBM SPSS Statistics (version 18; IBM Corporation, Armonk, NY, USA).

## 3. Results

### 3.1. Patients characteristics

This study screened 10,864 PwMS. After applying exclusion criteria, 4,984 PwMS were included (Fig 1). The average age was 42.08 years (SD: 10.29), with a female-to-male ratio 3.4:1. Among these patients, 86.9% had relapsing-remitting MS (RRMS), 12.1% had secondary progressive MS (SPMS), and 1% had primary progressive MS (PPMS). The median EDSS during follow-up was 1.5 (IQR: 2.5). Refer to (Table 1) for more details.

**Table 1:** Characteristics of patients with MS.

Age; mean (SD)			42.08 (10.29)
Disease duration; mean (SD)			10.10 (5.55)
EDSS at onset; median (IQR)			2 (0.5)
EDSS at follow-up; median (IQR)			1.5 (2.5)
Sex; n (%)	Female		3859 (77.4)
	Male		1125 (22.6)
MS type; n (%)	RRMS		4330 (86.9)
	PMS	SPMS	604 (12.1)
		PPMS	50 (1.0)
Drug; n (%)	DMF		573 (11.5)
	Fingolimod		386 (7.7)
	GA		370 (7.4)
	Interferon A		1598 (32.1)
	Interferon B		494 (9.9)
	Natalizumab		39 (0.8)
	OCR		3 (0.1)
	Other		201 (4)
	RTX		1023 (20.5)
	TFM		283 (5.7)
	None		14 (0.3)

Abbreviations: SD: Standard Deviation, IQR: Interquartile Range, EDSS: Expanded Disability Status Scale, CIS: Clinically Isolated Syndrome, RRMS: Relapsing-Remitting Multiple Sclerosis, PPMS: Primary Progressive MS, SPMS: Secondary Progressive MS, DMF: Dimethyl fumarate, GA: Glatiramer Acetate, OCR: Ocrelizumab, RTX: Rituximab, TFM: Teriflunomide.

### 3.2. Annual incidence of MS

According to the results of this study, the average annual incidence of MS over the 11 years studied was 1.70 per 10,000 people each year. Generally, the highest average incidence rates of MS were observed in the western part of the city, with 1.81 for the NW and 1.79 for the SW, while the NE recorded 1.68 and the SE reported 1.51, indicating a relatively better situation. For more details, see (Table 2).

**Table 2:** Annual incidence of multiple sclerosis in Isfahan city (per 10,000 population).

Year	Overall	NE	NW	SE	SW	p-value
2011	1.69	1.50	1.68	1.91	1.58	0.461
2012	2.29	2.42	2.36	1.84	2.67	0.065
2013	1.58	1.66	1.72	1.32	1.60	0.355
2014	1.82	1.49	2.01	1.67	2.02	0.166
2015	1.90	1.85	2.19	1.59	1.85	0.116
2016	2.28	2.21	2.54	1.96	2.32	0.199
2017	1.95	1.77	1.91	1.80	2.43	0.136
2018	1.74	2.07	1.86	1.28	1.84	**0.022**
2019	1.47	1.35	1.64	1.45	1.31	0.498
2020	1.39	1.57	1.53	1.07	1.42	0.115
2021	1.42	1.64	1.56	1.12	1.34	0.618

Abbreviations: NE: Northeast, NW: Northwest, SW: Southwest, SE: Southeast.

### 3.3. Distribution of PM2.5

The results of the average annual PM_2.5_ levels are presented in (Table 3). Based on these findings, the average PM_2.5_ level over the 11 years was 41.99 µg/m^3^. During the study period, the SW recorded the highest PM_2.5_ level in 2011 at 82.62 µg/m^3^ and the lowest in 2018 at 22.81 µg/m^3^. The average PM_2.5_ levels in the south were 42.62 µg/m^3^, compared to 40.85 µg/m^3^ in the north. In the west, the average level was 42.0 µg/m^3^, while the east recorded 41.5 µg/m^3^. According to the WHO air quality guideline, the recommended annual mean PM_2.5_ concentration should not exceed 5 µg/m^3^. In this study, all regions exhibited levels far above this threshold, with an average annual concentration of 41.99 µg/m^3^, indicating chronic exposure to unhealthy air.

**Table 3:** Annual average of PM_2.5_ in Isfahan city.

Year	Average 24-h PM2.5 – Mean (SD)- (μg/m^3^)					p-value
	Overall	NE	NW	SE	SW	
2011	59.21 (33.56)	42.21 (19.87)	54.19 (20.36)	57.71 (27.80)	82.62 (41.74)	**<0.001**
2012	44.17 (38.14)	45.68 (30.48)	44.76 (29.75)	42.86 (24.80)	43.39 (61.44)	**<0.001**
2013	64.21 (44.67)	51.30 (30.06)	78.68 (48.66)	52.50 (32.75)	74.36 (47.85)	**<0.001**
2014	51.05 (25.48)	45.99 (22.30)	51.62 (21.20)	56.35 (33.11)	48.52 (27.34)	**<0.001**
2015	37.52 (16.74)	36.68 (16.65)	37.88 (13.66)	38.52 (21.03)	36.52 (14.67)	**0.002**
2016	30.51 (11.77)	31.11 (12.36)	31.55 (11.82)	29.88 (11.58)	28.26 (10.45)	**<0.001**
2017	31.13 (17.51)	31.66 (18.80)	31.91 (17.17)	30.99 (18.33)	28.98 (13.85)	**0.024**
2018	26.47 (14.97)	28.66 (17.87)	26.46 (14.81)	25.46 (12.71)	22.81 (10.93)	**<0.001**
2019	39.83 (101.98)	33.34 (23.71)	28.72 (24.28)	78.47 (213.68)	25.80 (24.02)	**<0.001**
2020	40.13 (32.41)	47.37 (36.30)	36.23 (28.25)	29.83 (20.90)	36.37 (32.16)	**<0.001**
2021	37.71 (53.64)	41.76 (62.82)	41.55 (49.12)	34.73 (30.42)	32.83 (40.25)	**<0.001**
Overall	39.33 (38.75)	37.43 (33.93)	40.54 (31.15)	39.13 (53.01)	40.24 (36.91)	**<0.001**

Abbreviations: NE: Northeast, NW: Northwest, SW: Southwest, SE: Southeast, μg/m^3^: micrograms per cubic meter of air

### 3.4. Incidence Rate Ratio and PM2.5

This study investigated the association between MS incidence rate and PM_2.5_ level using three models. The first model analyzed the incidence rate with the PM_2.5_ level of the same year, the second examined the cumulative PM_2.5_ level with a one-year lag time, and the third assessed cumulative PM_2.5_ levels with a lag time of three years. The results presented in (Table 4) indicate that in the NW region of the city, the cumulative PM_2.5_ level with a one-year lag time significantly increased (IRR: 1.013, CI: 1.003–1.023, p-value: 0.008). According to the cumulative model with a 3-year lag time, an increase in PM_2.5_ level significantly elevated the IRR of MS in all four urban areas (IRR: 1.027, CI: 1.022–1.031, p-value < 0.001).

**Table 4:** Incidence rate ratio in different part of Isfahan.

	Model I		Model II		Model III	
	IRR (95% CI)	p-value	IRR (95% CI)	p-value	IRR (95% CI)	p-value
Overall	0.993 (0.987-0.999)	**0.035**	1.004 (0.997-1.010)	0.295	1.027 (1.022-1.031)	**<0.001**
NE	0.975 (0.960-0.991)	**0.003**	0.995 (0.983-1.007)	0.433	1.026 (1.16-1.037)	**<0.001**
NW	0.994 (0.988-1.001)	0.061	1.013 (1.003-1.023)	**0.008**	1.024 (1.016-1.031)	**<0.001**
SE	0.996 (0.981-1.011)	0.578	0.999 (0.996-1.002)	0.525	1.028 (1.17-1.039)	**<0.001**
SW	0.998 (0.993-1.003)	0.465	1.003 (0.988-1.018)	0.681	1.033 (1.028-1.039)	**<0.001**

Abbreviations: IRR: Incidence Rate Ratio, NE: Northeast, NW: Northwest, SW: Southwest, SE: Southeast.

**Fig 2:**
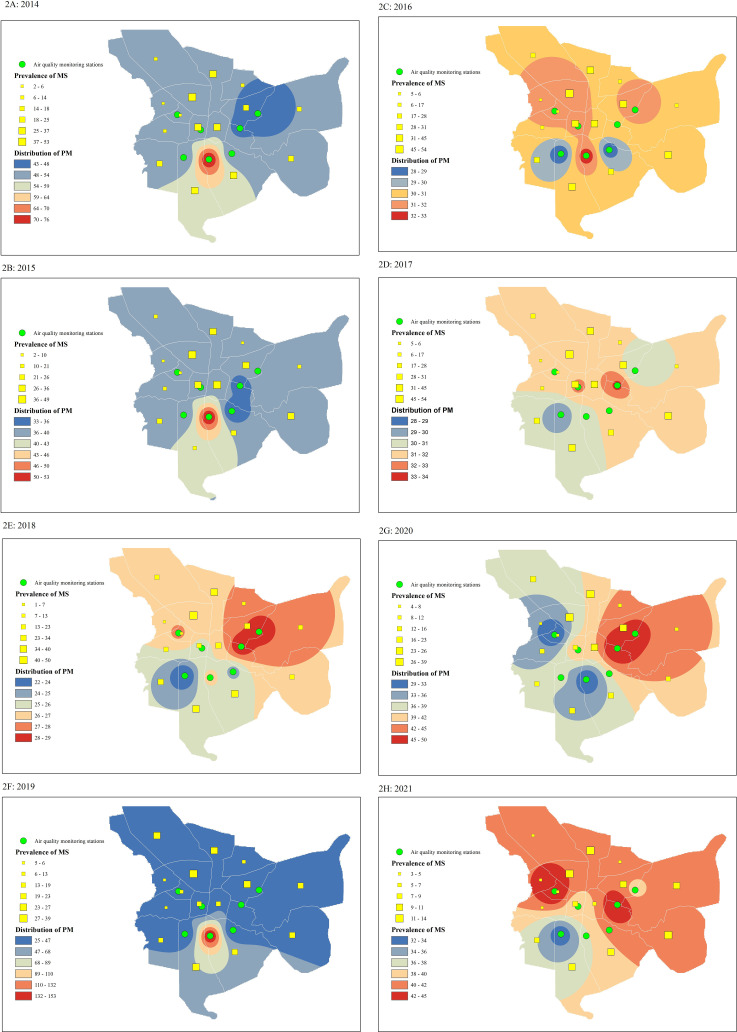
This figure illustrates the prevalence of multiple sclerosis across all fifteen urban areas, accompanied by the distribution of PM_2.5_, as represented on a GIS map. A-H based on years.

Please refer to (Fig 2) regarding the association between PM_2.5_ levels and the prevalence of MS. This figure indicates that in areas with consistently high PM_2.5_ levels, a higher prevalence rate of MS has been observed. The results shown in the figure were obtained through analysis using a Geographic Information System (GIS).

### 3.5. PM2.5 level and Disability in PwMS

In this study, PM_2.5_ levels were evaluated in relation to patients’ EDSS using common data obtained from the MUI database and the Kashani and Hakim MS Clinic. The results suggested a positive correlation between elevated PM_2.5_ levels and higher EDSS at the first relapse; however, this correlation was statistically significant only in the SW area (r: 0.053, p-value: 0.006).

According to the results presented in Table 5, the increase in EDSS during follow-up, compared to disease onset, which reflects disease progression, showed a positive correlation with PM_2.5_ levels. This correlation was statistically significant in the SE area for people with relapsing-remitting MS (PwRRMS) with (r: 0.173, p-value: 0.015). For people with progressive MS (PwPMS), significant correlations were observed in the NE, NW, and SW areas, with (r: 0.498, p-value: 0.013), (r: 0.244, p-value: 0.047), and (r: 0.589, p-value: 0.001), respectively. However, when analyzing all urban areas collectively, the correlation between the increase in EDSS and PM_2.5_ levels was statistically significant only in PwPMS (r = 0.321, p < 0.001). Refer to Table 5 for more information.

**Table 5:** Spearman correlation between PM_2.5_ and EDSS.

		Overall		NE		NW		SE		SW	
		r	p-value	r	p-value	r	p-value	r	p-value	r	p-value
EDSS at onset		0.013	0.669	0.041	0.573	0.021	0.670	0.035	0.580	**0.053**	**0.006**
EDSS at follow-up		−0.004	0.908	−0.061	0.400	−0.067	0.175	0.097	0.124	0.455	0.936
Delta EDSS	Total MS	0.013	0.672	0.024	0.738	0.044	0.374	**0.131**	**0.037**	0.040	0.575
	RRMS	0.047	0.174	0.013	0.875	0.046	0.415	**0.173**	**0.015**	0.034	0.673
	PMS	**0.321**	**<0.001**	**0.498**	**0.013**	**0.244**	**0.047**	0.180	0.248	**0.589**	**0.001**

Abbreviations: NE: Northeast, NW: Northwest, SW: Southwest, SE: Southeast, EDSS: Expanded Disability Status Scale, RRMS: Relapsing-Remitting Multiple Sclerosis, PMS: Progressive MS

## 4. Discussion

This study’s findings showed that long-term exposure to elevated levels of PM_2.5_ is associated with increase the risk of MS. Additionally, an increase in PM_2.5_ is associated with the progression of the disease.

### 4.1. Air pollution and MS in Isfahan

Isfahan, the city investigated in this study, has one of the highest prevalence rates of MS in the Middle East [25]. Various risk factors have been explored to understand the cause of this elevated prevalence, including air pollution [8], contamination of water and vegetables with heavy metals [33], vitamin D deficiency [6], genetics [34], and geographic latitude [35]. However, no single risk factor alone can fully account for the high prevalence of MS in Isfahan, and the high prevalence of MS is likely attributed to a combination of these risk factors. The findings of this study show that the incidence of MS in Isfahan is not homogenous, with higher prevalence rates observed in the southern and western areas compared to the northern and eastern (Fig 2).

Air pollution, a significant challenge in Isfahan, is probably a key risk factor contributing to the increased prevalence of MS in this city. Based on this study, the SW area experiences the highest levels of PM_2.5_, recorded at 82.62 µg/m^3^ in Isfahan. Factors contributing to this pollution in SW include fine dust and winds from the west, activities from large industries in the west of the province, including large steel and petrochemical industries, and major highways situated to the west and southwest of Isfahan.

However, the average PM_2.5_ level across all urban areas of Isfahan was significantly above the permitted limit, averaging 41.99 µg/m^3^. According to the U.S. Environmental Protection Agency’s National Ambient Air Quality Standards (NAAQS) [36], PM_2.5_ levels ≤ 12 µg/m^3^ are considered safe, 12–38 µg/m^3^ pose a moderate risk, and levels above 38 µg/m^3^ are classified as high risk. Based on these definitions, the level of PM_2.5_ in Isfahan during the 11 years of follow-up was classified as high risk for 8 years and moderate risk for 3 years. Consequently, PM_2.5_ levels were never within the safe range in any area of Isfahan throughout this study.

### 4.2. Is PM2.5 linked to incidence rate of MS?

According to the findings presented in Table 4, this study did not find a significant association between the average annual PM_2.5_ levels and the annual incidence rate of MS for the same year. This suggests that high pollution levels may not have an immediate impact on increasing the incidence rate of MS. However, after applying cumulative models, three-year cumulative pollution levels in each area were correlated with an increased risk of MS (Table 4, Model III). These results indicate that while PM_2.5_ levels may not affect MS incidence in the short term, long-term exposure could be a critical risk factor contributing to the high incidence of MS in Isfahan.

Although previous studies did not consider the cumulative effects of PM_2.5_, they frequently reported similar findings, supporting our results. For example, a study by Tateo et al., conducted in the Province of Padua, Italy, from 1998 to 2015, found that long-term exposure to high levels of PM_2.5_ can increase the risk of MS [9]. Additionally, the study by Scartezzini et al. remarked that long-term exposure to moderate levels of PM_2.5_ has a greater risk of MS than short-term exposure to high levels of PM_2.5_ [22]. However, the study by Bai et al., which investigated the correlation between PM_2.5_ and the MS incidence in Toronto, found no significant results [37]. The non-significant results were probably due to the lack of investigation into the cumulative association between PM_2.5_ levels and the annual incidence of MS. Further, Toronto, with an average PM_2.5_ level of 14.4 µg/m^3^, may not be an ideal location for studying the correlation between high PM_2.5_ levels and the incident rate of MS [37,38]. Based on these results, PM_2.5_ at safe levels may not significantly impact the incidence of MS. Considering that the average PM_2.5_ level in Isfahan is nearly five times higher than in Toronto and nine times above safe levels, the differing results between these two studies are understandable. Furthermore, geographical factors in Toronto, such as wind patterns, rain, latitude, and nearness to the ocean, may also contribute to the insignificant results.

### 4.3. Role of PM_2.5_ in MS pathology

PM_2.5_ consists of a carbon core complexed with various chemical compounds, including heavy metals, nitrogen oxides, sulfur oxides, and other pollutants, depending on the sources of industrial activities [39,40]. PM_2.5_ may contribute to the pathology of MS through two primary mechanisms [41].

1. PM_2.5_ can enter the bloodstream via the respiratory system, leading to the upregulation of CCR6 (Chemokine receptor 6), which facilitates the transportation of Th17 cells across the BBB [42]. Recent studies have identified Th17 as one of the primary autoaggressive lymphocytes involved in MS pathogenesis [43,44].
2. PM_2.5_ can stimulate dendritic cells (DCs) in the respiratory system to secrete pro-inflammatory cytokines, such as IL-1β, IL-6, and IL-23, which subsequently promote the differentiation of T cells into Th17 cells [41,44].

In addition to Its autoaggressive roles in MS, Th17 has aryl hydrocarbon receptors. These receptors can increase Th17 activity when they come into contact with hydrocarbons present in PM_2.5_ [41,45]. These mechanisms can lead to chronic and persistent inflammation in the respiratory system, CNS, and lymphoid organs. Long-term inflammation may increase susceptibility to autoimmune disorders, including MS [46].

In addition, PM_2.5_ can act as an oxidative stressor [47] and contribute to mitochondrial destruction [48]. Given the critical role of mitochondria in transmitting nerve signals and supporting neuronal health [49], their destruction can play a significant role in the pathogenesis of MS [50]. It may also induce PwMS to be more susceptible to neurodegeneration [50]. Furthermore, previous studies have shown that air pollution interacts as an activator with the HLA DRB1*15:01 allele, potentially increasing the risk of MS in individuals genetically predisposed to autoimmune disorders [51,52].

Increased air pollution may indirectly contribute to a higher incidence rate of MS. For example, increasing levels of air pollution can reduce UV light and lead to lower vitamin D levels [53]. Previous studies have identified vitamin D deficiency as a significant MS risk factor. Moreover, rising air pollution is associated with a higher prevalence of depression, which is a psychological risk factor for MS [20].

In addition, molecular studies indicate that PM2.5 acts as an oxidative stressor by disrupting the blood-brain barrier (BBB). These particles activate NADPH oxidase pathways, generating reactive oxygen species (ROS) both directly and indirectly via mitochondrial dysfunction [54]. The resulting oxidative stress may accelerate neurodegeneration and demyelination [55]. PM2.5 also upregulates matrix metalloproteinases, particularly MMP-9, which degrade tight junction proteins and compromise BBB integrity [56]. This increased permeability allows autoreactive lymphocytes to infiltrate the CNS, exacerbating neuroinflammation [57].

### 4.4. PM2.5 and MS exacerbation

Another aim of this study was to examine the relationship between disability levels of PwMS and atmospheric PM_2.5_ concentration. According to the results presented in (Table 5), higher PM_2.5_ levels were positively correlated with the severity of disability during the first relapse of patients, indicating that MS relapses become more severe when PM_2.5_ levels are elevated. However, this correlation was statistically significant only in the SW of Isfahan. Findings related to Delta EDSS, which reflects disease progression, and PM_2.5_ levels, indicate a positive correlation between elevated PM_2.5_ and disease progression in PwPMS. In other words, PwPMS living in more polluted areas experience faster disease progression and more severe clinical symptoms compared to those in cleaner air environments.

Previous studies have reported similar findings. For example, a study by Oikonen et al. in Finland showed that the risk of MS relapse on the most polluted days was four times higher than on unpolluted days (OR: 4.143) [21]. Ashtari et al. found a positive correlation between air pollution and EDSS. They also found that higher air pollution is linked to longer recovery times after relapses in PwMS [20]. Furthermore, Bergamaschi et al. indicated that for each 30 µg/m^3^ increase in PM_2.5_, the risk of a new T2-enhanced lesion in PwMS rises by 86% [53].

### 4.5. Strengths and limitations

The novelty of this study is that it uses a three-year lag time to demonstrate the association between cumulative air pollution and the incidence rate of MS. Another strength of this study is its population-based design, which included all patients living in Isfahan without selection bias, and the use of pollution data from seven monitoring stations covering different urban areas of the city. However, one of the limitations of the study was the lack of access to patients’ smoking status as a potential confounding variable. Previous research, including that by Elgabsi et al., suggests that smoking may be a minor confounder due to its differing nature from air pollution [58]. Other limitations include the heterogeneous distribution of PM_2.5_ monitoring centers and the lack of air pollution data available before 2011. Additionally, we were unable to include climate data such as temperature and precipitation due to inconsistencies between satellite records and local meteorological reports, as well as incomplete records for parts of the study period. This may have indirectly affected pollution patterns but could not be reliably assessed. Other potential residual confounding factors (e.g., diet, socioeconomic status, healthcare access), reliance on EDSS as the sole measure of disease progression, and challenges in establishing definitive causal relationships due to multiple dependent variables should be considered. Population-based cohort studies with longer follow-up periods are recommended.

## 5. Conclusion

This study concluded that although short-term exposure to PM_2.5_ cannot be associated with an increased risk of MS, it likely plays a significant role as a risk factor in long-term exposure. Furthermore, increasing levels of PM_2.5_ may be directly associated with disease progression, particularly in PwPMS.

## Supporting information

S1 File The data used in this study are provided in the supplementary 1 file. (RAR)
